# Algebraic Persistent Fault Analysis of SKINNY_64 Based on S_Box Decomposition

**DOI:** 10.3390/e24111508

**Published:** 2022-10-22

**Authors:** Xing Fang, Hongxin Zhang, Danzhi Wang, Hao Yan, Fan Fan, Lei Shu

**Affiliations:** 1 School of Electronic Engineering, Beijing University of Posts and Telecommunications, Beijing 100876, China; 2 Beijing Key Laboratory of Work Safety Intelligent Monitoring, Beijing University of Posts and Telecommunications, Beijing 100876, China; 3 Beijing Key Laboratory of Work Safety Intelligent Monitoring, School of Electronic Engineering, Beijing University of Posts and Telecommunications, Beijing 100876, China; 4 Beijing Microelectronics Technology Institute, Beijing 100076, China

**Keywords:** S_box, decomposition, persistent fault, algebraic equation, key residual entropy

## Abstract

Algebraic persistent fault analysis (APFA), which combines algebraic analysis with persistent fault attacks, brings new challenges to the security of lightweight block ciphers and has received widespread attention since its introduction. Threshold Implementation (TI) is one of the most widely used countermeasures for side channel attacks. Inspired by this method, the SKINNY block cipher adopts the S_box decomposition to reduce the number of variables in the set of algebraic equations and the number of Conjunctive Normal Form (CNF) equations in this paper, thus speeding up the algebraic persistent fault analysis and reducing the number of fault ciphertexts. In our study, we firstly establish algebraic equations for full-round faulty encryption, and then analyze the relationship between the number of fault ciphertexts required and the solving time in different scenarios (decomposed S_boxes and original S_box). By comparing the two sets of experimental results, the success rate and the efficiency of the attack are greatly improved by using S_box decomposition. In this paper, We can recover the master key in a minimum of 2000s using 11 pairs of plaintext and fault ciphertext, while the key recovery cannot be done in effective time using the original S_box expression equations. At the same time, we apply S_box decomposition to another kind of algebraic persistent fault analysis, and the experimental results show that using S_box decomposition can effectively reduce the solving time and solving success rate under the same conditions.

## 1. Introduction

With the development of Internet of Things (IoT) and chip technology, the security of information is becoming a key concern for people in practical production and life, and cryptography is gradually gaining popularity. In a resource-constrained environment, some lightweight block ciphers [1] have emerged in order to improve the security of information. They have the advantages of simple structure, high efficiency, and easy implementation. Common lightweight block ciphers include PRESENT, GIFT, SKINNY [2], LED, etc. These encryption ciphers are all substitution-permutation network (SPN) structures, where S_box substitution as a nonlinear operation plays a crucial role in the security of the ciphers. Therefore, it is important to conduct research on lightweight block ciphers and their S_boxes.

Fault attack, as a common side channel analysis method, has been receiving attention and research from scholars and experts since its inception. The main ways of fault attack [3] are voltage fault attack, electromagnetic fault attack, laser fault attack [4,5], temperature fault attack, etc. The purpose of these approaches is to alter the surroundings of the working cryptographic chip and change the encryption result by injecting an abnormal state. This concept was put forward by Boneh et al. on the RSA-CRT (Rivest-Shamir-Adleman China Remainder Theorem) algorithm in 1996. Fault attacks can also be divided into three types: transient faults, persistent faults, and permanent faults. In 2018, Zhang Fan et al. proposed a persistent fault analysis method. Their research injects faults into the S_box lookup tables of AES, PRESENT, and other algorithms, which makes errors occur when accessing the lookup tables, thus generating fault ciphertexts. The master key can be recovered by analyzing this fault injection method and the fault ciphertexts.

As a masking method, Threshold implementation (TI) [6,7,8] is a kind of side-channel attack countermeasure against first-order Differential Power Analysis (DPA) [9]. This method was first proposed by Nikova et al. in 2006. In 2011, Poschmann et al. proposed an algebraic decomposition method that decomposes the cubic S_box into two quadratic S_boxes [10]. This method was originally proposed to reduce the mask component, reduce circuit complexity, and reduce additional hardware resources. By this method, the original S_box algebraic system of equations [11] can be equivalently replaced by a new system of two algebraic equations.

### 1.1. Related Works

The current common analysis methods for persistent fault [12,13] are mainly classical persistent fault analysis (PFA) [14,15], enhanced persistent fault analysis (EPFA) [16], and persistent fault-Based collision analysis (PFCA) [17]. For the SKINNY block cipher, classical persistent fault analysis cannot recovery the master key. EPFA requires 1500–1600 fault ciphertexts to recover the master key. When using the PFCA method, we need plaintext selection depending on the algorithm structure and a large number of fault ciphertexts. This method requires a more complex application scenario compared to other methods. In order to drastically reduce the number of fault ciphertexts required by the attack, we try to introduce algebraic analysis methods into the persistent fault analysis.

In our previous research, we performed an algebraic fault analysis [18] based on S_box decomposition for the SKINNY block cipher. By performing the S_box decomposition, we can represent the algebraic characteristics of the S_box using a smaller number of CNF equations and variables. The experimental results show that the experimental efficiency is substantially improved after S_box decomposition. Therefore, we try to introduce this method when performing algebraic persistent fault analysis to improve the speed and success rate of solving.

### 1.2. Our Contributions

In this paper, we propose algebraic persistent fault analysis methods for the SKINNY block cipher based on S_box decomposition. This is first work that combines S_box decomposition methodology and algebraic analysis to recover the master key of the SKINNY cipher under the condition of a persistent fault in S_box. Our main contributions are as follows.

- For the SKINNY block cipher, its S_box is the four-in-four-out type, and the output four-bit value can be represented by an algebraic equation of the four-bit input value. When there is a fault in the S_box lookup table, the original algebraic equation cannot represent the output result, and an algebraic representation using the changed set of algebraic equations is required. We give the distribution of the number of variables in the algebraic equations of S_box by traversing all possible single faults in the S_box;
- In this paper, we propose an algebraic persistent fault analysis method based on known plaintexts(KP-APFA) with all rounds encryption. The attack is first attempted using the original faulty S_box algebraic expression. The experimental results show that the attack cannot complete key-recovery within the specified time;
- To achieve key-recovery of the SKINNY cipher, we introduce the S_box decomposition method and combine it with the KP-APFA method to analyze the SKINNY cipher, which can solve the key in 2000 swith at least 11 pairs of plaintext and faulty ciphertext. This reduces the number of fault samples by more than 100 times compared to the EPFA method;
- A constraint-based algebraic persistent fault analysis method was proposed by Zhang Fan et al. In this paper, the S_box decomposition is combined with this method (referred to as SD-APFA), and the experimental results show that the solving speed and the success rate of solving in the specified time are improved, and the best case can improve the solving speed by more than 10 times. In addition, the relationship between key residual entropy, fault depth, and number of faults is further investigated in this paper.

The remainder of this article is organized as follows. We describe the algorithmic structure of SKINNY in Section 2. Afterward, the persistent attack on s_box and the known-plaintext APFA method are presented in Section 3. We introduce the S_box decomposition methodology of SKINNY and simulation experiments of KP-APFA based on S_box decomposition in Section 4. In Section 5, we introduce the methodology of S_box decomposition to APFA (SD-APFA) and provide the attack results of different methods in several scenarios. Meanwhile, we further investigate the relationship between key residual entropy, fault depth, and the number of faulty ciphertexts. We give the experimental setup and results in Section 6, followed by the Conclusions in Section 7.

## 2. Algorithmic Description of SKINNY

The SKINNY block cipher is a lightweight AES-like tunable block cipher with a novel SPN structure, proposed by Beierle et al. at CRYPTO 2016. SKINNY is a class of tunable block cipher with tunable key framework, which is divided into six different versions according to the tunable key size and block length. In this paper we choose the most common version SKINNY_64_64 as the research object.

**Figure 1:**
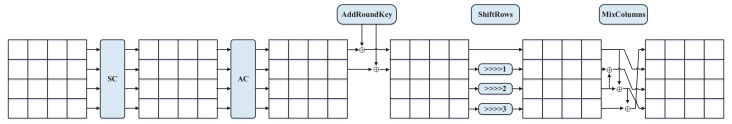
The structure of SKINNY_64.

Each encryption round of the SKINNY block cipher includes operations such as Subcells, Addconstants, AddroundTweakey, Shiftrows, and Mixcolumns. The single-round encryption process is shown in Figure 1. The number r of rounds to be performed during encryption depends on the block and tweakey sizes. For the SKINNY_64_64 version of the block cipher, its number of encryption rounds are 32.

- Subcells
  Subcells are the only non-linear operation in the entire encryption process. The hexadecimal notation of this S_box is given by the following Table 1.

**Table 1:** S_box lookup table of SKINNY.

X	0	1	2	3	4	5	6	7	8	9	a	b	c	d	e	f
S[X]	c	6	9	0	1	a	2	b	3	8	5	d	4	e	7	f

The S_box is a four-in and four-out type, and the four-bit values of output are related to the input four-bit values. Let the input of S_box be $x_{3}\|x_{2}\left\|x_{1}\right\|x_{0}$ and the output of S_box be $y_{3}\|y_{2}\left\|y_{1}\right\|y_{0}$, the algebraic relationship between them can be expressed by the following algebraic equations:
  (1) $$\begin{array}{cc} Y(x_{3},x_{2},x_{1},x_{0}) & =(y_{3},y_{2},y_{1},y_{0})\\ y_{0} & =x_{1}+x_{2}+x_{3}+x_{0}x_{1}+x_{0}x_{2}+x_{0}x_{3}+x_{1}x_{3}+x_{0}x_{1}x_{2}+x_{1}x_{2}x_{3}\\ y_{1} & =x_{0}+x_{3}+x_{0}x_{1}+x_{1}x_{2}+x_{1}x_{3}+x_{2}x_{3}+x_{1}x_{2}x_{3}\\ y_{2} & =1+x_{1}+x_{2}+x_{3}+x_{1}x_{2}\\ y_{3} & =1+x_{0}+x_{2}+x_{3}+x_{2}x_{3} \end{array}$$
  Observation of Equation (1) reveals that the original S-box algebraic equation uses a total of eight quadratic and quadratic+ variables, which are $x_{0}x_{1}$, $x_{0}x_{2}$, $x_{0}x_{3}$, $x_{1}x_{2}$, $x_{1}x_{3}$, $x_{2}x_{3}$, $x_{0}x_{1}x_{2}$, $x_{1}x_{2}x_{3}$.

- Addconstants
  The constants of the SKINNY block cipher are generated through a 6-bit affine LFSR (Linear Feedback Shift Register), whose state is updated by following definition:
  (2) $$\left(rc_{5},rc_{4},rc_{3},rc_{2},rc_{1},rc_{0}\right)\leftarrow =\left(rc_{4},rc_{3},rc_{2},rc_{1},rc_{5}⊕rc_{4}⊕1\right)$$
  The initial value of these 6 bits is set to 0, which are updated before use in a given round. The bits from the LFSR are arranged into a 4 × 4 array (only the first column of the state is affected by the LFSR bits):
  (3) $$\left[\begin{array}{cccc} c_{0} & 0 & 0 & 0\\ c_{1} & 0 & 0 & 0\\ c_{2} & 0 & 0 & 0\\ 0 & 0 & 0 & 0 \end{array}\right]$$
  with $c_{2}=0\times 2$, $\left(c_{0},c_{1}\right)=(rc_{3}\|rc_{2}\|rc_{1}\|rc_{0},0\|0\|rc_{5}\left\|rc_{4}\right)$.

- AddRoundTweakey
  The first and second rows of all tweakey arrays are extracted and bitwise exclusive-oredto the cipher internal state, respecting the array positioning. The specific subkey generation method can be found in Ref. [2].

- ShiftRows
  This operation can be represented as a permutation. A permutation P is applied on the cells positions of the cipher internal state cell array: for all 0≤i≤15, the operation can be showed as P=[0,1,2,3,7,4,5,6,10,11,8,9,13,14,15,12].

- MixColumns
  This operation can mix each column by multiplication. The matrix M of the multiplication is shown as follows:
  (4) $$\left[\begin{array}{cccc} 1 & 0 & 1 & 1\\ 1 & 0 & 0 & 0\\ 0 & 1 & 1 & 0\\ 1 & 0 & 1 & 0 \end{array}\right]$$

## 3. Persistent Fault Injection in S_Box

The cryptographic world has never stopped attacking the SKINNY block cipher since this cipher’s inception. The SKINNY has good security properties and can bring security to information under resource-constrained conditions. Since Zhang Fan et al. proposed the persistent fault analysis method, persistent fault analysis on lightweight cryptographic algorithms has become one of the attack trends. Persistent fault analysis is the injection of faults into the S_box lookup table, which causes errors to occur when accessing specific S_box units. Selecting the appropriate fault injection point is one of the main focuses of the study.

Injecting a single fault in the S_box gives a total of 16×15=240 possibilities. Our study traverses all possibilities and gives the changed set of S_box equations. By counting the number of intermediate variables in the changed set of equations, the least number of cases is selected for building the equation.

Algorithm 1 is used to iterate through all individual persistent faults of the S_box and generate a system of algebraic fault equations for the S_box and calculate the number of higher-order variables in the system of equations. It is worth stating that the values of the following statistics are the number of variables that are not duplicated. A statistical table of the number of quadratic and quadratic+ variables in the original S_box for different persistent fault scenarios is given in Table 2.
**Algorithm 1:** Pseudocode for calculating the number of higher-order variables in a system of equations for an S_box
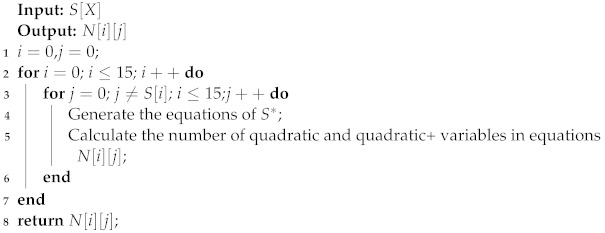


According to previous research experience, the more quadratic and above variables in the S_box equations, the slower the solving speed will be. Therefore, when injecting fault into the look-up table, in order to improve the solution efficiency, the number of intermediate variables in the modified equation sets should be as small as possible. When the persistent fault is injected into the S_box lookup table and makes F[2] = 9 into F[2] = A, the number of quadratic and quadratic+ variables is 7, and its corresponding equation expression becomes:

(5) $$\begin{array}{cc} Y(x_{3},x_{2},x_{1},x_{0}) & =(y_{3},y_{2},y_{1},y_{0})\\ y_{0} & =x_{2}+x_{3}+x_{0}x_{2}+x_{0}x_{3}+x_{1}x_{2}+x_{0}x_{1}x_{3}+x_{0}x_{1}x_{2}x_{3}\\ y_{1} & =x_{0}+x_{1}+x_{3}+x_{2}x_{3}+x_{0}x_{1}x_{2}+x_{0}x_{1}x_{3}+x_{0}x_{1}x_{2}x_{3}\\ y_{2} & =1+x_{1}+x_{2}+x_{3}+x_{1}x_{2}\\ y_{3} & =1+x_{0}+x_{2}+x_{3}+x_{2}x_{3} \end{array}$$

Algorithm 2 is used to generate the faulty ciphertext. *P* and *f* are the inputs to the Algorithm 2, representing plaintext and fault, respectively. The output of Algorithm 2 is the fault ciphertext $C^{*}$. The function of Algorithm 2 is equivalent to simulating a persistent fault injection experiment.
**Algorithm 2:** The fault ciphertext generation of SKINNY_64
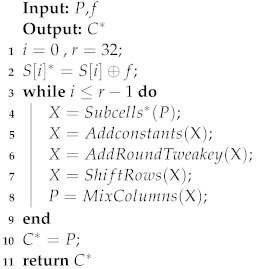


In Algorithm 3, we give a pseudocode for algebraic persistent fault analysis based on known plaintext, referred to as KP-APFA. The input *N* in the algorithm represents the number of faults, i.e., the number of plaintext and fault ciphertext pairs. The output in the algorithm is the solving time. For the SKINNY block cipher, there is a constant algebraic relationship between the subkey and the master key for each round. We represent the faulty encryption process in the form of algebraic equations and convert all the useful information into the form of a system of CNF equations. Then, all the CNF equations are combined to perform the key solution in the $T_{sol}=RunAPFA\left(\right)$ means using CryptoMiniSAT to get the time of key-recovery.

We use the CryptoMiniSAT for key-recovery, which requires converting the algebraic equations into the form of CNF equations. For correct encryption, each round of S_box can be represented with 192 variables and 480 CNF equations, the round-constant is generated by a 6-bit affine LFSR, it can be represented with 6 variables and 6 CNF equations. Considering the operations of the Addconstants, Subkey, AddRoundTweakey, ShiftRows, and MixColumns, each round of them can be represented with 320 variables and 320 CNF equations.

Equation (5) gives the set of algebraic equations for the S_box after the persistent fault makes F[2] = 9 into F[2] = A. For this faulty encryption, each round of fault S_box can be represented with 176 variables and 464 CNF equations. Considering the operations of the Addconstants, AddRoundTweakey, ShiftRows, and MixColumns, each round of them can be represented with 256 variables and 256 CNF equations.

Use the above method to build a set of equations for persistent algebraic fault analysis. Experiments were set up using 16, 18, 20, and 30 random plaintexts, respectively, and for the purpose of discussing the generality of the experiments, 50 samples were randomly generated for each set of experiments. The maximum solving time is set to 1 h in the experiment, and the solution is judged to fail if it exceeds 1 h. The results of the study show that all samples cannot complete the recovery of the key within the specified time, therefore, the key-recovery cannot be completed by using the original S_box directly. We need to improve the expression of S_box algebraic equations.
**Algorithm 3:** The KP-APFA for SKINNY_64
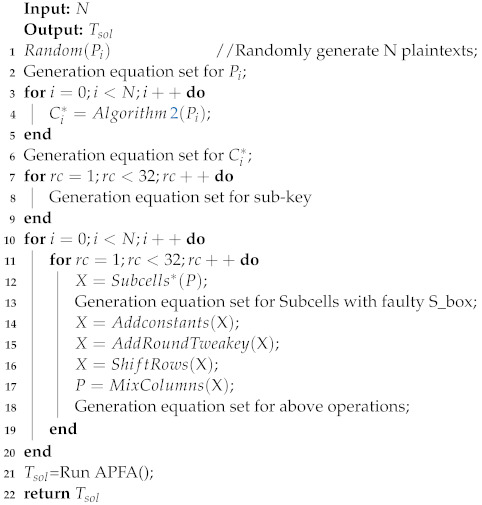


## 4. S_Box Decomposition of SKINNY

**Figure 2:**
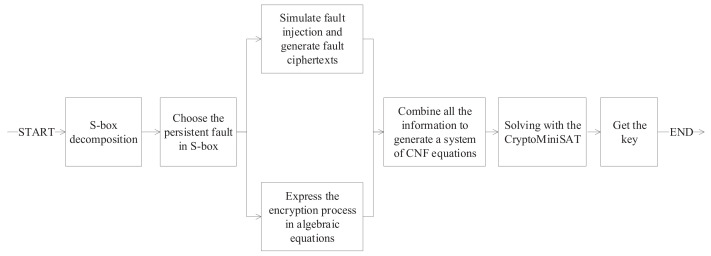
The flowchart of algebraic persistent fault analysis based on S_box decomposition.

This section gives the general flow of algebraic persistent fault analysis based on S_box decomposition. Firstly, the S_box in the target algorithm is decomposed and a suitable decomposition scheme is selected. Then, all possible individual S_box persistent faults are traversed and a suitable fault injection scheme is selected. The key-recovery is finally done by simulating the fault injection to generate the faulty ciphertexts and build the cryptographic algebraic system of equations and other useful information using the CryptoMiniSAT. The specific flowchart is given in Figure 2.

**Figure 3:**
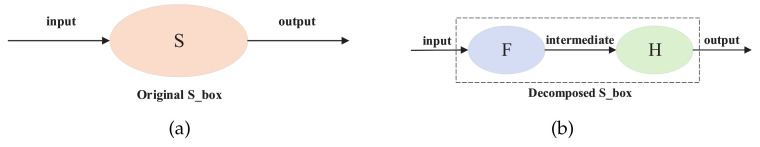
Original S_box and decomposed S_box. (**a**) original S_box (**b**) decomposed S_box.

In our previous research on the SKINNY_64 for algebraic fault analysis, we found that the use of S_box decomposition can greatly improve the speed of the key-recovery. We can decompose the original cubic S_box into two quadratic S_boxes to reduce the number of CNF clauses in the set of algebraic equations and the number of quadratic and quadratic+ intermediate variables introduced due to the nonlinear operation S_box. A schematic diagram of the decomposition is given in Figure 3. In 2011, Poschmann et al. proposed a technique to decompose a cubic S_box function into two quadratic functions.This relation can be expressed by the following equations S(X)=H(F(X)), where $S,F,H:GF{\left(2\right)}^{4}\to GF{\left(2\right)}^{4}$. Considering the input and output of G(X) as 4-bits vectors X=(x,y,z,w) and $F\left(X\right)=(f_{0}\left(X\right),f_{1}\left(X\right),f_{2}\left(X\right),f_{3}\left(X\right))$. Each $f_{i}$, as a quadratic Boolean function, can be represented in ANF as the following equation, where $a_{i},a_{ij}$ are the binary coefficients of the Boolean function:

(6) $$f_{i}\left(x,y,z,w\right)=a_{0}+a_{1}x+a_{2}y+a_{3}z+a_{4}w+a_{12}xy+a_{13}xz+a_{14}xw+a_{23}yz+a_{24}yw+a_{34}zw$$

As discussed in literature [10], in order to reduce the overall search space for the two decomposed functions of F and H, the following two facts were used in this paper:

- Rewriting S(X)=H(F(X)) as $S(F^{-1}\left(X\right)=H\left(X\right)$, one needs to search only for all possible quadratic functions for G(X). This is then used to compute the other quadratic functions F(X) as $S(F^{-1}\left(X\right))$.
- Rewriting S(X)=H(F(X)) as $S\left(X\right)=H^{{}^{\prime }}\left(F^{{}^{\prime }}\left(X\right)\right)$ where $F^{{}^{\prime }}\left(X\right)=F\left(X\right)+F\left(0\right)$ and $H^{{}^{\prime }}\left(X\right)=H\left(X+F\left(0\right)\right)$. We assume that G(0)=0 and get the other decompositions directly by substituting 15 nonzero values for G(0). Therefore, we only need to vary the 10 nonconstant coefficients in the ANF and the search space is reduced to ${\left(2^{10}\right)}^{4}=2^{40}$.

With the above refinements, the actual quadratic Boolean equation used for the search is shown below.

(7) $$f_{i}\left(x,y,z,w\right)=a_{1}x+a_{2}y+a_{3}z+a_{4}w+a_{12}xy+a_{13}xz+a_{14}xw+a_{23}yz+a_{24}yw+a_{34}zw$$

The following steps were implemented in order to compute the desired optimized quadratic Boolean functions for F and H:

- We first iterate through all combinations of coefficients of function $f_{i}$. For each combination of coefficients we iterate through all possible values of *X* and check whether the Boolean equation is balanced. If the combination is balanced we then add it to a set B for all possible coefficients, otherwise it is discarded;
- The corresponding F(X) equation is calculated iteratively for each balanced coefficient in the set B in a group of four;
- Check whether the computed F(X) is a quadratic permutation or not. If yes, we compute the H(X), otherwise we discard it;
- Check whether the computed H(X) is a quadratic function or not. If yes, add both the F(X) and H(X) functions to the set P of all the possible decompositions, otherwise discard them. By doing the above, we can obtain the possible decomposition of 19,862 pairs;
- Considering 15 other possible constant terms, we finally get 317,777 possibilities for the S-box decomposition of the SKINNY block cipher using the above steps.

**Figure 4:**
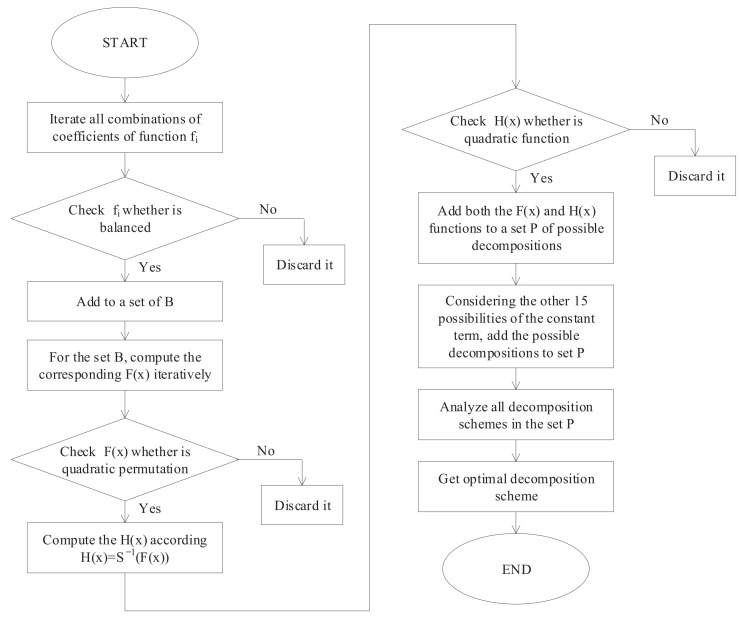
The flowchart of S_box decomposition.

Figure 4 gives the flowchart of the decomposition of the cubic S_box into two quadratic S_boxes.

We followed the method provided in the literature [16] for censoring and finally obtained the optimal decomposition scheme. The selected F(X) and H(X), satisfying all the three TI requirements, namely Correctness, Non-Completeness, and Uniformity, are shown in Table 3.

The ANFs of first S_box can be represented by Equation (8).

(8) $$\begin{array}{cc} F(w,z,y,x) & =(f_{3},f_{2},f_{1},f_{0})\\ f_{0} & =x+z+xw+w\\ f_{1} & =y\\ f_{2} & =y+yz+z+w\\ f_{3} & =z \end{array}$$

The ANFs of second S_box can be represented by Equation (9).

(9) $$\begin{array}{cc} H(w,z,y,x) & =(h_{3},h_{2},h_{1},h_{0})\\ h_{0} & =y+yz\\ h_{1} & =x+xy+w\\ h_{2} & =1+z\\ h_{3} & =1+x \end{array}$$

By looking at the set of Equations (8) and (9) we find that by decomposing the S_box, the number of quadratic and quadratic+ intermediate variables introduced is changed from the original 8 to 2 + 2. Next, we perform the persistent fault injection for the first S_box. The number of quadratic and quadratic+ intermediate variables is calculated by traversing all fault injection cases. The specific experimental results are shown in Table 4. According to the traversal results given in Table 4 we can find that when the fault is injected into S[F], the fault S_box has the least number of introduced intermediate quadratic and quadratic+ variables. Combining the original S_box and the decomposed S_box1 with the fault-injection intermediate variable traversal table, the S-box decomposition is used to minimize the number of quadratic and quadratic + variables and the number of CNF equation sets that can be used to express algebraic persistent fault analysis with minimal CNF equations. Next, an example is used to illustrate. Injecting fault in S[F] causes S[F] = A to become S[F] = E in the first S_box after decomposing, and for the whole encryption process, this change causes S[F] = F to become S[F] = B.

Table 5 is the decomposed fault S_box1. The corresponding algebraic equation for the fault S_box is shown below.

(10) $$\begin{array}{cc} F^{*}\left(w,z,y,x\right) & =(f_{3},f_{2},f_{1},f_{0})\\ f_{0} & =x+z+xw+w\\ f_{1} & =y\\ f_{2} & =y+yz+z+w+xyzw\\ f_{3} & =z \end{array}$$

For the original S_box, this persistent fault is equivalent to the following expression.

Table 6 is the faulty original S_box. The corresponding algebraic equation for the fault of the original S_box is shown in Equation (11).

(11) $$\begin{array}{cc} Y(x_{3},x_{2},x_{1},x_{0}) & =(y_{3},y_{2},y_{1},y_{0})\\ y_{0} & =x_{1}+x_{2}+x_{3}+x_{0}x_{1}+x_{0}x_{2}+x_{0}x_{3}+x_{1}x_{3}+x_{0}x_{1}x_{2}+x_{0}x_{1}x_{2}x_{3}\\ y_{1} & =x_{0}+x_{3}+x_{0}x_{1}+x_{1}x_{2}+x_{1}x_{3}+x_{2}x_{3}+x_{1}x_{2}x_{3}\\ y_{2} & =1+x_{1}+x_{2}+x_{3}+x_{1}x_{2}+x_{0}x_{1}x_{2}x_{3}\\ y_{3} & =1+x_{0}+x_{2}+x_{3}+x_{2}x_{3} \end{array}$$

We use the decomposing S_boxes algebraic equations and the original S_box algebraic equations for persistent algebraic fault analysis experiments, respectively.

**Figure 5:**
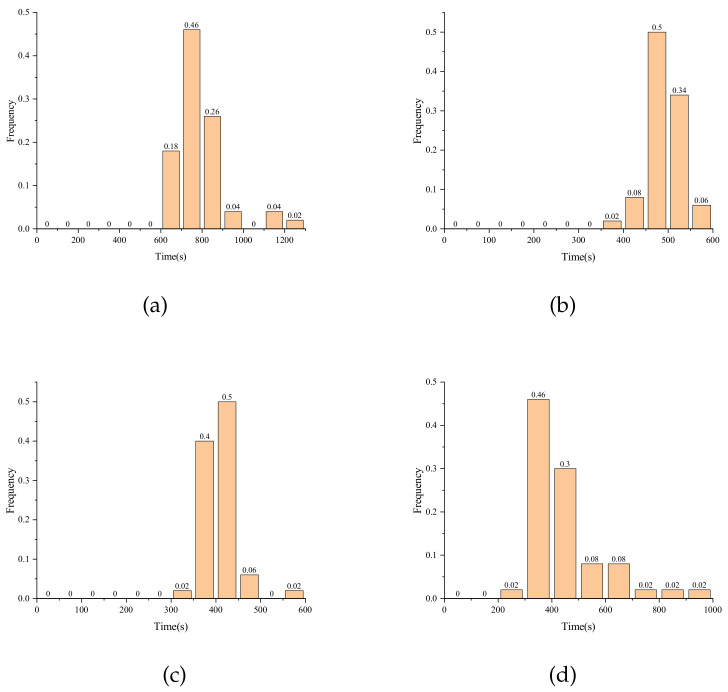
Distribution of solving time under different scenarios of new S_boxes. (**a**) N = 30; (**b**) N = 20; (**c**) N = 18; (**d**) N = 16.

First, we set the number of faulty plaintexts to 30, 20, 18, and 16, respectively. Fifty samples are randomly generated in each scenario, and the two methods are compared in experiments under the same fault conditions. The experiment sets the maximum solving time of the solver to 1 h, and the attack is judged to have failed after 1 h. Table 7 gives the average solving time and success rate of the two methods for different scenarios. The experimental results show that the key can be solved in effective time after using S_box decomposition, while all experiments cannot complete the key-recovery in effective time when using the original S_box expression. Figure 5 gives a histogram of the distribution of the solving time when using S_box decomposition in different scenarios, where the horizontal coordinates represent the solving time and the vertical coordinates represent the frequency. When the number of faulty ciphertexts is 30, the average solving time is 784.9 s. When the number of faulty ciphertexts is reduced to 20 and 18, the average solving time decreases. This may be due to the information redundancy caused by the larger number of algebraic equations when the number of faulty ciphertexts is large, and thus the solving time is longer than when the number of faulty ciphertexts is 20 and 18. As the number of faulty ciphertexts decreases further, the average solving time increases. When the number of faulty ciphertexts is 16, the average solving time reaches 450.6 s. From the histogram, we can find that 48% of the samples can be solved for the key within 400 s.

**Figure 6:**
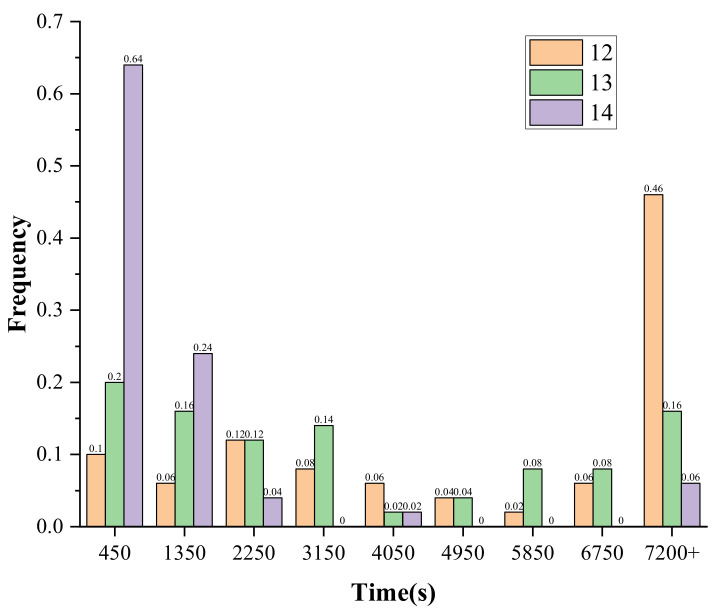
Histogram comparison of solving the time distribution under different scenarios of SKINNY_64.

The Table 8 shows that when the setting time is 2 h and the number of ciphertexts is 12, the success rate is 54%. When the maximum solving time is relaxed to 10 h, the success rate of the solving for the same experimental samples reaches 92%, where the shortest solving time is 459.5 s. In our study, in order to explore the minimum number of ciphertexts that can be used to achieve key-recovery, the number of ciphertexts is further reduced to 11, 10 sets of samples are randomly selected, the maximum solving time is set to 10 h, and the attack is judged to failure after 10 h. The experimental results show that 50% of the samples can be solved within the specified time, and the shortest solving time is 1930.9 s. Figure 6 gives the histogram of the solving time distribution for the number of faulty ciphertexts of 14, 13, and 12. From Figure 4, we can find that as the number of faults decreases from 14 to 12, the number of samples that complete key-recovery within 450 s decreases from 64% to 10%, while the number of samples that do not complete key-recovery within 7200 s increases from 6% to 46%.

## 5. Applying Another Persistent Algebraic Analysis of SKINNY

A method for persistent algebraic fault analysis [19] was proposed by Fan Zhang et al. in 2022, and the SKINNY block cipher was studied in the literature. Inspired by this literature, the relationship between the number of faulty ciphertexts, fault depth, and key residual entropy is analyzed on the basis of the original research in my paper. Our paper adopts the method of S_box decomposition instead of the original S_box, and analyzes the differences between the two methods in the same scenario. In the literature [19], Zhang Fan et al. proposed a constraint-based method to solve the problem of unsolvability due to information redundancy in full-round encryption. The method is briefly described in the following.

Suppose the original value of the fault S_box $S^{\prime }$ to be V. In the *r*-th(1≤r≤32) round function, $X^{r}$ is the input of the *r*-th (1≤r≤32) round, $Y^{r}=MC\left(SR\left(K^{r}⊕\left(AC\left(SC^{\prime }\left(X^{r}\right)\right)\right)\right)\right)$. $Y^{r}$ contains the information of both $SC^{\prime }\left(X^{r}\right)$ and $K^{r}$, which means that we can use $Y^{r}$ to add new constraints to $K^{r}$, as shown in Equation (12).

(12) $$\left.\begin{array}{c} SC^{\prime }\left[X_{i}^{r}\right]\neq V\\ Y^{r}=MC\left(SR\left(K^{r}⊕\left(AC\left(SC^{\prime }\left(X^{r}\right)\right)\right)\right)\right)\\ MC^{-1}\left(SR^{-1}\left(Y^{r}\right)\right)=SC^{\prime }\left(X^{r}\right)⊕AC^{-1}\left(K^{r}\right) \end{array}\right\}\Rightarrow \tilde{K_{i}^{r}}\neq \tilde{Y_{i}^{r}}⊕V,0\leq i\leq 15$$

In Equation (12), $X_{i}^{r}$ and $\tilde{Y_{i}^{r}}$ are the 4 bits of $X^{r}$ and $MC^{-1}\left(SR^{-1}\left(Y^{r}\right)\right)$, respectively. $\tilde{K_{i}^{r}}$ are the 4 bits of $AC^{-1}\left(K^{r}\right)$ and *V* is a constant. Based on this inequality relation, we establish constraints so as to reduce the key search space. The reader can check the specific steps in the literature [19].

Algorithm 4 gives the pseudo-code for the SKINNY block cipher based on S_box decomposition for algebraic persistent fault analysis. The input of the algorithm *N* is the number of faulty ciphertexts, FD is the fault depth, and the output $T_{sol}$ is the solving time of the solver. First the algorithm randomly generates *N* plaintexts and uses Algorithm 2 to get the set of faulty ciphertexts, then all faulty ciphertexts are transformed into CNF equations. Next, we express the relationship between each round of subkeys and the master key as an algebraic equation and transform it into the form of CNF equations. Based on the input fault depth, we perform an algebraic representation of the FD rounds encryption process. We use two decomposed S_boxes instead of the original S_boxes, and transform the algebraic equations for each of these two S_boxes. Similarly, we translate the information about Addconstants, AddRoundTweakey, ShiftRows, MixColumns and constraints into the form of algebraic equations. Finally, we associate all the sets of CNF equations and use $T_{sol}$ = Run SD−APFA() for key-recovery. It is worth stating that the solver is set to work for 1 h, and the attack is considered to fail if it is exceeded. Fifty samples are randomly generated for each set of experiments, and the average solving time and solving success rate are calculated in Table 9.

Table 9 gives the average solving time and the success rate of the solving within the specified time for the two methods for different scenarios. N represents the number of faulty ciphertexts and FD represents the depth of the fault. Under different scenarios, we randomly generated 50 samples and set the maximum solving time to 1 h. When N = 30 and FD = 4, the use of the new S_box algebraic representation can improve about 18 times over the original method. When the number of faulty ciphertexts is 20, 18, 17, 16, and 14, both methods are able to recover the keys within the specified time. At the same time, the solving speed is improved using the new S_box algebraic equations compared to the original method. When the number of faulty ciphertexts is 13 and 12, the solving success rate and average solving time using the new method are better than the original method. From the above experimental results, it is shown that the solving speed and solving success rate can be effectively improved by using the new S_box algebraic expression method.
**Algorithm 4:** The SD-APFA for SKINNY_64
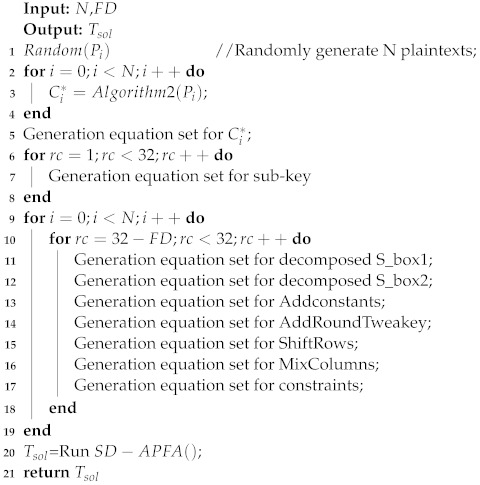


When using the solver for solving the system of CNF equations, multiple solutions exist because many intermediate variables are introduced and these intermediate variables have relatively few constraints. As for the key, each faulty ciphertext can constrain the key, so the value of the key may be the same. We take the first solution solved by the solver as the object of study and analyze the relationship between the solved key and the correct key. If they are the same, the key is determined to be unique, and if they are different, the key residual entropy is determined to be greater than 0. In other words, the value of the key is not unique.

We analyze and discuss the relationship between the number of faulty ciphertexts, fault depth, the key residual entropy, and the success rate of solving in a specified time under the new method. From Table 10, we find that when the number of faulty ciphertexts is 30 and the fault depth is 6, the first solution obtained by all 50 samples is equal to the correct key, and the key residual entropy is determined to be 0 in this experiment. Meanwhile, when the fault depth is reduced to 4, only 84% of the samples obtain the first solution equal to the correct key, while the remaining 16% obtain the first solution with a different value from the correct key. As the number of faulty ciphertexts decreases, there is such a relationship between the fault depth and the residual entropy of the key. When the fault depth is shallow, the key residual entropy cannot be 0, at the same time, the fault depth must be increased to satisfy that the key residual entropy is 0.

**Figure 7:**
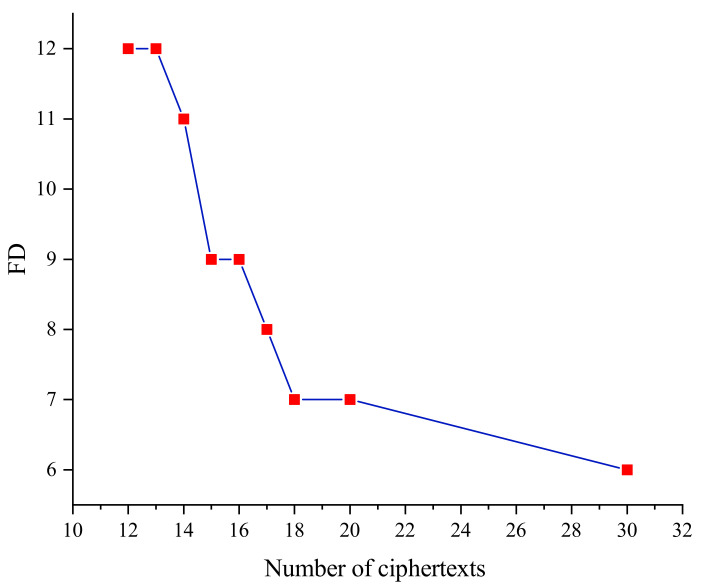
Relationship between the number of ciphertexts and fault depth (entropy = 0).

Figure 7 gives the relationship between the number of faulty ciphertexts and the minimum fault depth when the key residual entropy is 0 in the SKINNY_64 block cipher. We can find that the minimum fault depth decreases gradually as the number of fault ciphertexts increases, in other words, there is an inverse relationship between the minimum fault depth and the number of fault ciphertexts. When the number of faulty ciphertexts is small, if the fault depth is shallow, there are fewer constraints on the key information, so there are more keys satisfying the algebraic equations and the first solution given by the solver is different from the correct key. When deepening the fault depth, the generated set of algebraic equations has more constraints on the key, which improves the possibility of the key being the unique solution.

## 6. Experimental Setup and Results

In this section, we will introduce our setup of the experiments and the comparison results with a variety of existing methods.

### 6.1. Experimental Setup

In our experiment, we simulate the fault injection experiment via software, and use the CryptoMiniSAT v5.8.0 as the solver to solve the algebraic equations using Ubuntu 18.04.5 on Windows. We implement the experiments on a PC that has 16 GB memory and Intel(R) Core(TM) i5-9500 CPU at 3 GHz. The operating system is a 64 bit Windows 10.

### 6.2. Results

Table 11 provides a variety of existing methods to persistent fault analysis of SKINNY_64. The relationship between precondition, fault depth, minimum number of faults, and key residual entropy for different methods is given in the table.

**Figure 8:**
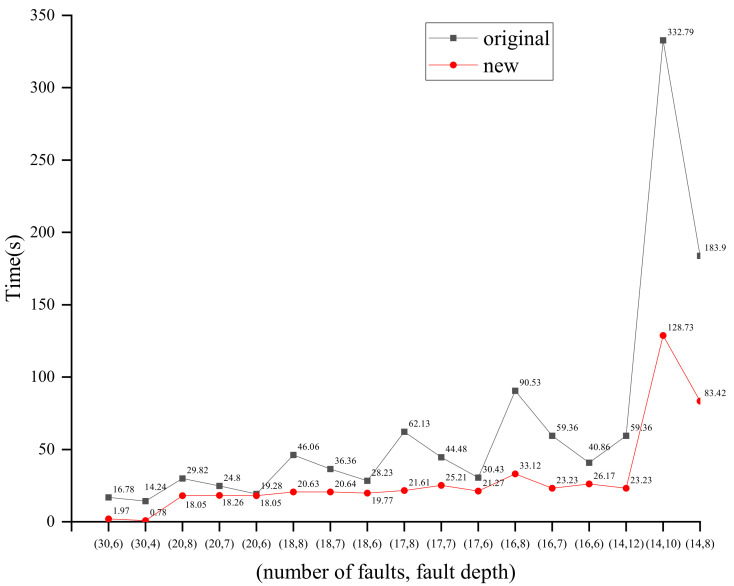
Comparison of the solving time with the two methods in different scenarios.

From Table 11 we can see that by using the PFA method, the key cannot be fully recovered because the fault depth is 1. The residual entropy of the key is 32. The EPFA method uses multiple rounds of faulty information and can recover the entire key. A minimum of 1500–1600 faulty ciphertexts are required to complete key-recovery. In this paper, the proposed KP-APFA based on S_box decomposition can complete key-recovery using a minimum of 11 faults, and unlike other methods, plaintexts need to be provided. The SD-APFA method proposed in this paper based on APFA also requires a minimum of 10 fault ciphertexts, but we can find that it improved the solving speed and solving success rate in the same attack scenarios as in Figure 8 and Table 9.

## 7. Conclusions

In this paper, we combine S_box decomposition methods commonly found in the field of threshold implementation with algebraic analysis. A more suitable persistent fault injection scheme is found from the perspective of algebraic analysis. Under the condition of known plaintexts, we have conducted several sets of experiments on the KP-APFA method based on S_box decomposition, and the average solving time and solving success rate are given for a different number of faults. The key-recovery can be completed within 2000s using at least 11 faults. Meanwhile, this paper also combines the method of S_box decomposition with the APFA method, and the proposed SD-APFA method has been significantly improved in both solving speed and success rate. Meanwhile, we discuss the relationship between key residual entropy, number of faults, and fault depth in different methods. In summary, the simplification of the system of equations using the S_box decomposition technique to achieve the S_box substitution operation is beneficial to improve the solving speed of the CryptoMiniSAT, thus improving the attack efficiency of the attacker in persistent fault analysis. In future work, we will apply and generalize this approach on other lightweight block ciphers.

## Figures and Tables

**Table 2:** Numbers of quadratic and the above variables under different faults in the original S_box.

	0	1	2	3	4	5	6	7	8	9	a	b	c	d	e	f
F[0]	11	11	11	11	11	11	10	11	11	11	10	11	—	8	11	11
F[1]	11	11	11	11	11	10	—	8	11	11	11	11	11	11	11	11
F[2]	10	10	10	10	10	10	10	10	9	—	**7**	10	10	10	10	9
F[3]	—	9	10	9	10	10	10	10	10	10	10	10	10	10	10	10
F[4]	8	—	9	10	10	10	10	9	10	10	10	9	10	10	10	10
F[5]	10	10	10	10	10	10	10	10	10	10	—	8	10	10	10	10
F[6]	9	8	—	8	8	9	9	9	9	9	9	9	9	9	9	9
F[7]	9	9	9	9	9	9	9	9	9	9	8	—	9	9	9	9
F[8]	9	11	10	—	11	11	11	11	11	10	11	11	11	11	11	11
F[9]	11	11	11	11	11	11	11	11	—	10	11	11	11	11	11	11
F[a]	10	10	10	10	10	—	8	10	10	10	10	10	10	10	10	10
F[b]	10	10	10	10	10	10	10	10	10	10	10	10	10	—	10	10
F[c]	10	10	10	10	—	10	10	9	10	10	10	10	10	10	9	10
F[d]	10	10	10	10	10	10	10	10	10	10	10	10	10	10	—	10
F[e]	9	9	9	9	8	9	9	—	9	9	9	9	9	9	9	9
F[f]	9	9	9	9	9	9	9	9	9	9	9	9	9	9	9	—

**Table 3:** The decomposed S_boxes.

X	0	1	2	3	4	5	6	7	8	9	a	b	c	d	e	f
F[X]	0	1	6	7	d	c	f	e	5	4	3	2	9	8	b	a
H[X]	c	6	d	5	8	3	9	0	e	4	f	7	a	1	b	2

**Table 4:** Numbers of quadratic and the above variables under different faults in decomposed S_box1.

	0	1	2	3	4	5	6	7	8	9	a	b	c	d	e	f
	0	1	2	3	4	5	6	7	8	9	A	B	C	D	E	F
F[0]	—	10	11	11	10	11	11	11	11	11	11	11	11	11	11	11
F[1]	9	—	9	9	9	9	9	9	9	9	9	9	9	9	9	9
F[2]	8	8	7	8	8	8	—	8	8	8	8	8	8	8	8	8
F[3]	6	6	6	6	6	6	6	6	—	6	6	6	6	6	6	6
F[4]	7	7	7	7	7	7	7	7	7	6	7	7	6	—	7	7
F[5]	6	6	6	6	6	6	6	6	6	6	6	6	—	6	6	6
F[6]	5	5	5	5	5	5	5	5	5	5	5	4	5	5	5	—
F[7]	4	4	4	4	4	4	4	4	4	4	4	4	4	4	—	4
F[8]	8	8	8	8	7	—	8	8	8	8	8	8	8	8	8	8
F[9]	6	6	6	6	—	6	6	6	6	6	6	6	6	6	6	6
F[a]	6	6	6	—	6	6	6	6	6	6	6	6	6	6	6	6
F[b]	4	4	—	4	4	4	4	4	4	4	4	4	4	4	4	4
F[c]	5	5	5	5	5	5	5	5	4	—	5	5	5	5	5	5
F[d]	4	4	4	4	4	4	4	4	—	4	4	4	4	4	4	4
F[e]	4	4	4	4	4	4	4	4	4	4	4	—	4	4	4	4
F[f]	3	3	3	3	3	3	3	3	3	3	—	3	3	3	3	3

**Table 5:** The decomposed fault S_box1.

X	0	1	2	3	4	5	6	7	8	9	a	b	c	d	e	f
F${}^{*}$[X]	0	1	6	7	d	c	f	e	5	4	3	2	9	8	b	e

**Table 6:** The faulty original S_box.

X	0	1	2	3	4	5	6	7	8	9	a	b	c	d	e	f
S${}^{*}$[X]	c	6	9	0	1	a	2	b	3	8	5	d	4	e	7	b

**Table 7:** The results of the algebraic persistent fault analysis on SKINNY_64 under different scenarios.

N( Number)	$T_{\mathrm{ave}}$ (Seconds) of Original S_Box	$T_{\mathrm{ave}}$ (Seconds) of New S_Boxes	Success Rate of Original S_Box	Success Rate of New S_Boxes
30	-	784.9	0%	100%
20	-	496.2	0%	100%
18	-	409.6	0%	100%
16	-	450.6	0%	100%

**Table 8:** The results of the new S_boxes on SKINNY_64 under different scenarios.

N (Number)	$T_{\mathrm{ave}}$ (Seconds) of New S_Boxes	Success Rate of New S_Boxes
14	833.0	94%
13	2739.2	84%
12	3040.6	54%

**Table 9:** Comparison table of the two methods in different scenarios.

N	FD	$T_{\mathrm{ave}}$ (Seconds) of Original S_Box	$T_{\mathrm{ave}}$ (Seconds) of New S_Boxes	Success Rate of Original S_Box	Success Rate of New S_Boxes
30	6	16.78	1.97	100%	100%
30	4	14.24	0.78	100%	100%
20	8	29.82	18.05	100%	100%
20	7	24.80	18.26	100%	100%
20	6	19.28	18.05	100%	100%
18	8	46.06	20.63	100%	100%
18	7	36.36	20.64	100%	100%
18	6	28.23	19.77	100%	100%
17	8	62.13	21.61	100%	100%
17	7	44.48	25.21	100%	100%
17	6	30.43	21.27	100%	100%
16	8	90.53	33.12	100%	100%
16	7	59.36	23.23	100%	100%
16	6	40.86	26.17	100%	100%
14	12	59.36	23.23	100%	100%
14	10	332.79	128.73	100%	100%
14	8	183.90	83.42	100%	100%
13	12	1112.10	508.57	96%	100%
13	10	656.31	338.52	94%	98%
12	14	1972.54	847.60	44%	68%
12	12	1255.55	760.94	56%	72%
12	10	1334.56	956.65	76%	86%

**Table 10:** Table of the experimental results of new S_boxes under different scenarios.

N	FD	Key Residual Entropy = 0	Success Rate of New S_Boxes
30	6	100%	100%
30	4	84%	100%
20	8	100%	100%
20	7	100%	100%
20	6	96%	100%
18	8	100%	100%
18	7	100%	100%
18	6	96%	100%
17	8	100%	100%
17	7	98%	100%
17	6	86%	100%
16	9	100%	100%
16	8	96%	100%
16	6	76%	100%
14	12	100%	100%
14	10	98%	100%
14	8	88%	100%
13	12	100%	100%
13	10	98%	98%
12	14	68%	68%
12	12	72%	72%
12	10	76%	86%

**Table 11:** Comparison with existing persistent-fault-analysis on SKINNY_64.

Methods	Precondition	FD	Minimum Number of Faults	Key Residual Entropy
PFA	Ciphertext only	1	≈100	32
EPFA	Ciphertext only	4	1500–1600	0
APFA	Ciphertext only	6–14	10	0
KP-APFA (this paper)	Known plaintext/ciphertext	32	11	0
SD-APFA (this paper)	Ciphertext only	6-14	10	0

## Data Availability

If necessary, the data can be requested from the author of the communication.
